# Updates and reflections about the IJHPR, on the eve of its seventh year

**DOI:** 10.1186/s13584-017-0196-6

**Published:** 2017-12-28

**Authors:** Bruce Rosen, Avi Israeli

**Affiliations:** 1 Smokler Center for Health Policy Research, Myers-JDC-Brookdale Institute, JDC Hill, Jerusalem, Israel; 2 Hadassah – Hebrew University Medical School, Jerusalem, Israel; 3 Ministry of Health, Jerusalem, Israel

## Abstract

The Israel Journal of Health Policy Research (IJHPR) will soon be completing 6 years of publication. During this period, it has published well over 300 articles and has become a stable and vital part of Israeli health care. The number of IJHPR articles published annually has increased significantly over time, and the number of submissions has increased even more significantly. The journal is regularly drawing submissions from a very broad range of Israeli universities, hospitals, health plans, and other institutions, and from leading institutions abroad. The journal’s articles cover a wide spectrum of subjects related to health policy, as reflected in the diversity of the article collections that the journal has launched to date. True to its mission statement, the IJHPR is promoting “intensive intellectual interactions among scholars and practitioners from Israel and other countries regarding all aspects of health policy”.

The journal has been indexed in all of the leading international academic databases and its current impact factor (1.36) puts it in the third quartile in two key Web of Science subject categories. Several IJHPR articles have been accessed over 10,000 times and the IJHPR has become the predominant vehicle in which Israeli scholars are publishing articles with a health policy orientation. IJHPR articles are also figuring prominently in many course syllabi in Israel, and Israeli universities have incorporated IJHPR publications into assessments of faculty members for promotion. The journal’s success and progress are a part of a larger trend of greater reliance on data and analysis in health policymaking in Israel and the increasing prominence of health in the Israeli policy agenda and public discourse.

During the journal’s seventh year, its editors will be carrying out a serious assessment of the challenges and opportunities ahead and developing an updated plan for the journal’s development. In doing so, they will draw on data presented in this editorial as well as on in-depth discussions with those who have contributed to the journal’s success to date: it publisher, sponsor, and editorial board members, as well as a sample of authors, reviewers and readers. The expectation is that, by working together, it will be possible to take the journal to new heights.

## Introduction

The Israel Journal of Health Policy Research was launched in 2012 by the National Institute for Health Policy and Health Services Research. Based on several well attended international meetings held in Israel, and frequent collaboration of the rapidly growing community of Israeli health policy scholars with counterparts abroad, the editors and the National Institute recognized that the level of international interest in the Israeli health system was substantial, and well in excess of the norm for health systems in small countries. At the same time, they realized that, because of its small size, it is particularly important for Israeli health care to learn from the experience of other countries. They realized that a peer-reviewed journal could take this cross-national learning, and existing efforts to link policy and research within Israel, to a new level. Thus, as indicated in the journal’s mission statement “Israel Journal of Health Policy Research seeks to promote intensive intellectual interactions among scholars and practitioners from Israel and other countries regarding all aspects of health policy…. The ultimate aim of these intellectual interactions is to contribute to the development of health policy in Israel and around the world.”

The IJHPR will soon be completing its sixth year of publication and beginning its seventh. In both Biblical and academic traditions, after 6 years of intensive activity, the seventh year is championed as a time for reflection. At the IJHPR, we are also planning to use the upcoming seventh year as a time of summing up and looking to the future. Accordingly, in comparison with previous end-of-year editorials, we are using our 2016 editorial to provide a more comprehensive look at what the journal has accomplished to date. This will lay the groundwork for a serious assessment during 2017 of the challenges and opportunities ahead.

## Achievements

Perhaps first and foremost, the journal has become a stable and vital part of Israeli health care. When the journal was launched, there were many who had serious questions about the need for such a journal and whether it would attract a sufficient volume of high quality articles. Now, after 6 years in which the journal has published well over 300 articles, with at least one article published every month of its existence, those questions about viability have disappeared. Indeed, in the words of Orly Manor, the chairperson of the National Institute’s executive committee, “The National Institute takes great pride in the accomplishments of the IJHPR and it is an integral part of the National Institute’s program. The journal is also an integral part of the Israeli health care system, documenting its strengths as well as opportunities for improvement in policies and practices.”

The journal’s success and progress are a part of a larger trend of greater reliance on data and analysis in health policymaking in Israel and the increasing prominence of health in the Israeli policy agenda and public discourse. Related phenomena are the increased attention to health in the media, the growing number of health policy conferences, and the growing levels of attendance at these conferences. The journal has certainly benefited from these broader trends; we believe that the journal has contributed to them as well.

Health policy in general, and the IJHPR in particular, are also becoming more prominent in Israeli academia. IJHPR articles are figuring prominently in many course syllabi in Israel, and Israeli universities have incorporated IJHPR publications into assessments of faculty members for promotion. The journal has been indexed in all of the leading international academic databases, including PubMed, PubMed Central, Web of Science, Social Science Citation Index, and Scopus.

**Fig. 1:**
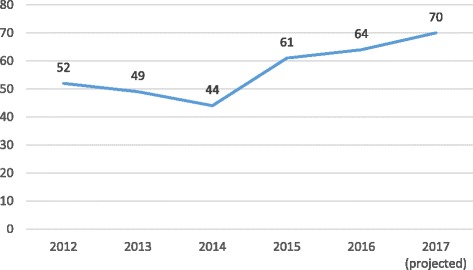
Articles published by year

As can be seen in Fig. 1, the journal has published over 60 articles in each of the past 3 years, a significant increase from an average of less than 50 during the journal’s first 3 years. Submissions are up even more, to over 120 per year, allowing the journal to become more selective and to raise its quality bar.

A related point is that the IJHPR has become the predominant vehicle in which Israeli scholars are publishing articles with a health policy orientation. In fact, the IJHPR accounts for over a third (52 out of 142) of the articles associated with Israeli universities that were published in 2015–6 in any of the 77 journals included in the Web of Science’s Health Policy and Services category.

**Fig. 2:**
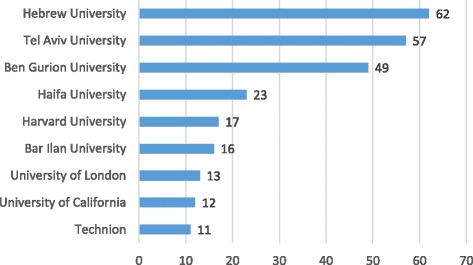
Universities associated with 10 or more IJHPR articles (Based on Web of Science, as of December 1, 2017)

Significantly, the journal is drawing submissions from a broad range of Israeli universities, as can be seen in Fig. 2. The three universities associated with the greatest number of publications are Hebrew University, Tel Aviv University, and the Ben-Gurion University of the Negev. It is important to note that many of these articles and authors are also associated with institutions other than universities, such as hospitals, health plans, research centers, and government agencies.^1^

Also worthy of note are the major contributions from three universities from outside of Israel: Harvard University, the University of London and University of California. These contributions are mostly in the form of commentaries, highlighting the international significance of empirical studies of Israeli health care. The IJHPR is also increasingly publishing original research articles and integrative articles written jointly by Israeli scholars and scholars from other countries. These contributions from abroad are a testimony to the high regard in which the Israeli health system, and its researchers, are held by leading international scholars.

We are also pleased that the journal is increasingly drawing high-quality submissions from professionals working in the government and in health care provider organizations. This reflects both the journal’s policy orientation and the growing involvement in research, analysis and academia among professionals in these organizations. The journal’s policy orientation also accounts for the important contributions from scholars in other countries who had recently served in high-level positions in their respective governments.

While institutions provide vital support for research and publication, in the end it is individuals who write the articles and select the journals to which they will submit them. And so, after 6 years, we feel the time is ripe to publicly acknowledge those individuals who have made particular contributions to the IJHPR’s growing corpus of intellectual holdings.

Table 1 lists the five IJHPR articles that have been accessed most often [1–5]. Leading the list is Carmel Shalev and Gabriele Werner-Felmayer’ 2012 article entitled “Patterns of globalized reproduction: Egg cells regulation in Israel and Austria”, with just over 20,000 accesses. The lead authors for the other articles on this list are Jonathan Weiner, Jochanan Benbassat, Manfred Green, and Nurit El-bar; three out of four of those articles have been accessed over 10,000 times. In the years ahead, we hope that the increasing use of article collections and social media will enhance the visibility of the many important articles published in the IJHPR, and increase the level of interest and engagement among readers from both Israel and abroad.

**Table 1:** Most accessed articles (Based on Biomed Central database, as of December 1, 2017)

Patterns of globalized reproduction: Egg cells regulation in Israel and Austria
Carmel Shalev and Gabriele Werner-Felmayer - 2012 (20,296)
Doctor-patient communication in the e-health era
Jonathan Weiner - 2012 (16,369)
The effect of clinical interventions on hospital readmissions: a meta-review of published metaanalyses
Jochanan Benbassat and Mark Taragin - 2013 (15,168)
Climate change and health in Israel: adaptation policies for extreme weather events
Manfred S Green, Noemie Groag Pri-or, Guedi Capeluto, Yoram Epstein and Shlomit Paz – 2013 (10,551)
Compassion fatigue, burnout and compassion satisfaction among family physicians in the Negev area
Nurit El-bar, Amalia Levy, Hedy S Wald and Aya Biderman – 2013 (9,612)

Table 2 lists the 10 IJHPR articles that have been cited most often, according to the Web of Science [1, 2, 5–12]. Leading the list, with 16 citations is “Compassion fatigue, burnout and compassion satisfaction among family physicians in the Negev area - a cross-sectional study” by Nurit El-bar et al.. This is followed by “The association between continuity of care in the community and health outcomes: a population-based study” by Jacob Dreiher et al., with 15 citations. Interestingly, the list of 10 most cited articles spans a very broad range of topics, disciplines and affiliated institutions – reflective of the journal as a whole.

**Table 2:** Most cited IJHPR articles (Based on Web of Science, as of December 1, 2017)

Cites	Year	Title and author
16	2013	Compassion fatigue, burnout and compassion satisfaction among family physicians in the Negev area - a cross-sectional study Nurit El-bar et al..........
15	2012	The association between continuity of care in the community and health outcomes: a population-based study Jacob Dreiher et al
14	2012	Which health technologies should be funded? A prioritization framework based explicitly on value for money Ofra Golan and Paul Hansen
14	2012	Community healthcare in Israel: quality indicators 2007–2009 Dena Jaffe et al
13	2012	Doctor-patient communication in the e-health era Jonathan Weiner
11	2012	Quality of online health information about oral contraceptives from Hebrew-language websites Yehuda Neumark et al
10	2015	Tobacco policy in Israel: 1948–2014 and beyond Laura Rosen and Maya Peled-Raz
10	2014	Israeli nurse practice environment characteristics, retention, and job satisfaction Freda Ganz et al
10	2013	The effect of clinical interventions on hospital readmissions: a meta-review of published meta-analyses Jochanan Benbassat and Mark Taragin
10	2012	Medical specialty considerations by medical students early in their clinical experience Charles Weissman et al

This breadth is also reflected in the journal’s growing list of article collections (also known as thematic series), which currently includes the following:

- Pharmaceutical policy issues
- Health economics
- Digital Health
- Health care equity
- Primary care
- Prioritization
- Health promotion and disease prevention
- The healthcare workforce
- Quality of care

These article collections are important tools for both research and teaching, and help our authors reach additional audiences. Of course, the range of topics covered by the journal is even broader, including such topics as health care and the elderly, medical education, and much more. We welcome suggestions of additional topics for article collections.

In recent years, the journal has been attracting and publishing more articles that take on major, national health policy issues. The topics covered include: reducing the burden of STI infections [13], Israel’s pediatric dental care reform [14], strategies for reducing health inequalities [15], tobacco control policy [10], educating the future Israeli medical workforce [16], drug shortages [17], medically-assisted reproduction [18], and vaccine policy [19]. In the absence of the IJHPR, some of these articles might well have been published in other journals and, in a few cases, the authors have frankly shared with us that their submissions had previously been rejected by other journals, usually because of perceived lack of relevance. However, in quite a few other cases, authors of broad policy articles have told us they never would have written the manuscripts if the IJHPR had not created an appropriate platform for their publication. They also noted that they found the input of IJHPR reviewers and commentators to be uniquely valuable, in combining both national and international perspectives, and in integrating in-depth familiarity with the Israeli policy process with methodological and conceptual expertise. We look forward to receiving more of these sorts of “big policy” papers in the future.

**Fig. 3:**
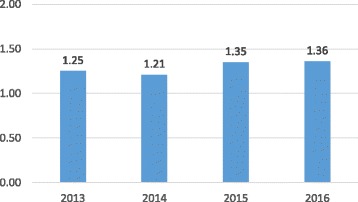
IJHPR impact factor

Data on the IJHPR’s Web of Science impact factor are presented in Fig. 3. The journal’s impact factor has been remarkably stable; no small accomplishment among new journals, which often “flame out” after a statistically impressive first year in the Web of Science. Still, we would like to see this statistic increase in the years ahead. Until now, we have consistently been in the third quartile of both the Health Policy & Services category (currently 47 of 77) and the Public, Environmental and Occupational Health category (currently 92 of 157). We are eager to move the journal into the second quartile as soon as possible with contributions of excellent manuscripts from experienced and new authors.

The data also suggest that the IJHPR has become a venue in which Israeli authors are publishing some of their best articles related to health policy. In 2015–6, 27,757 articles were published in the 77 journals in the Web of Science’s Health Policy & Services, and 142 of those articles are associated with an Israeli university. Despite the recency of publication, 9 of the 142 have already been cited five or more times, and 4 of those 9 were published in the Israel Journal of Health Policy Research.

## Appreciation of special contributions

The IJHPR recognizes that its success is due in large part to its authors – authors who have carried out important scholarly work, written it up, submitted a manuscript to the IJHPR, and stuck with us through the rigorous process of review and revision. We appreciate the contributions of all of our authors, and are especially appreciative of those who publish with us regularly. Sixteen Israeli scholars have published 4 or more articles in the IJHPR, and nine of them have published five or more articles in the IJHPR. As editors, we work hard to ensure that authors have a positive experience in their interactions with the journal, so “repeat business” of this sort is particularly gratifying.

The journal’s success is also due in large part to the work of hundreds of reviewers, from Israel and abroad. As editors, we repeatedly and consistently hear from our authors about how impressed they are with the quality of the reviews they receive and how appreciative they are of how the reviewers’ constructive input has helped them improve their papers. The names of all IJHPR reviewers for the years 2012 through 2016 can be found here; the list for 2017 is due to be published in early 2018.

Another group that has played a key role in the journal’s work is its editorial board, whose members are listed here. Editorial board members play a vital role in helping us identify reviewers and commentators, as well as undertaking a large number of reviews themselves. A special thanks goes to the journal’s associate editor, Steve Schoenbaum, for the tremendous amount of work he has put in to ensure the quality of our articles.

Another special thank you goes to Alik Aviram, who will soon be completing his tenure as scientific director of the National Institute, and who has been a major factor in the IJHPR’s success. Alik has been a regular source of wise counsel and encouragement. We wish Alik success in all his future endeavors and are delighted that he has informed us that he will now have more time available to help out with the journal.

We also wish to express our profound appreciation for the late Haim Doron, who for decades played a central role in the development of the Israeli health care system, and who also played a key role in the launching and development of the IJHPR. He was the Director-General of Clalit Health Services, Chairman of the National Institute’s Board of Directors, one of the fathers of primary care in Israel, and Chairman of the IJHPR’s editorial board. Professor Doron, who passed away in November 2017 at the age of 89, was throughout his long and productive life a visionary, a leader, and a mensch. He began talking about the need for an Israeli health policy journal over 15 years ago, and as such was probably the first significant figure in Israeli health care to do so. In appreciation of this, the journal’s primary care article collection will be dedicated to the memory of Professor Doron. May his memory be an ongoing inspiration for all of us.

## Looking to the future

As we look to the future, we hope that more and more of our readers will submit high quality manuscripts to the IJHPR. Articles published in the IJHPR have the potential to contribute significantly to improved health policymaking and population health, both in Israel and around the world.

In the year ahead, we will be exploring several new ways to use the journal to better link research and policymaking and to bring the international experience to bear on the Israeli policy development process. We also plan to use the journal’s seventh year to carry out a serious assessment of the challenges and opportunities ahead and to develop an updated plan for the journal’s development. In doing so, we will draw on the data presented here as well as in-depth discussions with all of the journal’s key partners and constituents. We look forward to working together in the effort to take the journal to new heights.

## Abbreviations

IJHPR: Israel Journal of Health Policy Research

## Footnotes

1 The chart does not include data on the number of articles associated with organizations other than universities because of inconsistencies in how these associations are recorded in the Web of Science.
